# Immunogenic Properties and Safety of a Quadrivalent Inactivated Subunit Adjuvanted Influenza Vaccine in Adults Aged 18 to 85 Years at the End of the COVID-19 Pandemic in the 2022–2023 Season

**DOI:** 10.3390/vaccines14020181

**Published:** 2026-02-14

**Authors:** Mikhail P. Kostinov, Aristitsa M. Kostinova, Sofia Iushkova, Lilia Gladkova, Anna Vlasenko, Yulia Dagil, Maria Kvasova, Anastasia Kameleva, Anastasia Kachnova, Irina Solovеva, Anna Khamidulina, Ekaterina Prutskova, Irina Mekhantseva, Natalia Andreeva, Valentina B. Polishchuk, Yvette Albahansa Mana, Anton M. Kostinov

**Affiliations:** 1 Department of Epidemiology and Modern Vaccination Technologies, I. M. Sechenov First Moscow State Medical University (Sechenov University), Moscow 119991, Russia; monolit.96@mail.ru (M.P.K.); aristica_kostino@mail.ru (A.M.K.);; 2 I. I. Mechnikov Research Institute of Vaccines and Sera, Moscow 105064, Russia; 3 Moscow Health Department, D. D. Pletnev City Clinical Hospital, Moscow 105077, Russia; 4 Federal State Budgetary Educational Institution of Higher Education, Samara State Medical University, The Ministry of Healthcare of the Russian Federation, Samara 443079, Russia; 5 National Research Center, Institute of Immunology of the Federal Medical Biological Agency of Russia, Moscow 115522, Russia; 6 Federal State Budgetary Educational Institution of Higher Education, Privolzhsky Research Medical University, Ministry of Health of the Russian Federation, Nizhniy Novgorod 603005, Russia; 7 Department of Clinical Immunology, Allergology and Adaptology, P. Lumumba Russian University of Peoples’ Friendship, Moscow 117198, Russia; 8 Department of Pediatrics, Institute of Medicine, Ecology and Physical Culture, Ulyanovsk State University, Federal State Budgetary Educational Institution of Higher Education, Ulyanovsk 432007, Russia; 9 Department of Internal Medicine, Karaganda Medical University, Karaganda 100012, Kazakhstan; 10 Department of Healthcare Management and Economics, The Chuvash State University Named After I. N. Ulyanov, Federal State Educational Establishment of Higher Professional Education, Cheboksary 428015, Russia; 11 Budgetary Institution “City Children’s Clinical Hospital”, Ministry of Health of the Chuvash Republic, Cheboksary 428000, Russia

**Keywords:** adults, SARS-CoV-2 infection, adjuvanted influenza vaccine, azoximer bromide, immunogenicity, influenza, vaccination

## Abstract

**Background**: SARS-CoV-2 infection has raised concerns about altered immune responses, creating a need to evaluate influenza vaccine performance in the post-COVID period. This study aimed to compare the immunogenicity and safety of a quadrivalent inactivated subunit adjuvanted influenza vaccine in adults aged 18–85 years during the 2022–2023 season. **Methods**: A total of 144 adults were enrolled: group 1, aged 18–59 years (*n* = 124), and group 2, aged 60–85 years (*n* = 20). All received a quadrivalent inactivated subunit adjuvanted vaccine containing 5 μg of each influenza antigen and 500 μg of Azoximer bromide. IgG antibodies to vaccine strains were measured at baseline and days 30–32 using the hemagglutination inhibition assay. Participants were actively monitored for adverse events by telephone. **Results**: The Geometric Mean Fold Increase (GMFI) met the efficacy criteria in both age groups (≥2.5 for 18–59 years and ≥2.0 for 60–85 years), with no significant differences. The seroprotection rate reached accepted thresholds for most strains but was below criteria for B/Victoria in the 18–59 group (48%) and for B/Phuket in the 60–85 group (35%). Significant between-group differences were observed for B/Victoria (*p* = 0.01) and B/Phuket (*p* = 0.007). Seroconversion met criteria for all strains in younger adults, but for older adults, it was insufficient for B/Phuket (20%, below the ≥30% threshold; *p* = 0.05 vs. 18–59 years). Local reactions occurred in 24.2% and systemic in 11.3% of younger adults; in older adults, in 20% and 15%, respectively. All resolved spontaneously within 1–3 days. **Conclusions**: The quadrivalent adjuvanted influenza vaccine demonstrated acceptable immunogenicity and safety in adults aged 18–85 years despite potential post-COVID immune alterations.

## 1. Introduction

Seasonal influenza vaccination is undoubtedly highly effective in preventing or reducing viral and bacterial infections in the population. Its effectiveness directly depends on many factors, such as the timely update of the influenza vaccine strain composition and its match with the circulating strains in a certain region, the start of vaccination campaigns and coverage levels among priority groups, the presence of current seasonal vaccination, and the natural circulation of viruses [1,2,3]. The technologies used in the production of immunobiological products that enhance the vaccine’s protective properties—especially for the elderly—are also of great importance in achieving effective influenza vaccination. Also, public awareness efforts (sociological aspects) aimed at improving vaccine perception are crucial. These efforts are part of a broader health culture and promote lifelong vaccine adherence.

Vaccine effectiveness often varies depending on age, sex, ethnicity, and genetics [4,5,6,7]. However, the recent SARS-CoV-2 pandemic posed additional challenges to immune system responses, including the development of post-vaccination specific immunity, which differed from patterns observed prior to the pandemic. According to data from a European multicenter study conducted in 2022–2023, the effectiveness of the influenza A(H3N2) vaccine was 36% [95%CI: 25–45] across all age groups, varying from 30% to 52% among those aged 15–64 and 0–14, respectively. For influenza A(H1N1)pdm09, vaccine effectiveness was 46% [95%CI: 35–56], ranging from 29% to 59%. For influenza B, effectiveness was 76% [95%CI: 70–81], ranging from 84% to 71% [8]. In the UK, at the end of the 2022/23 season, influenza vaccine effectiveness was found to be insufficient across all laboratory-confirmed cases in individuals over 65, most of whom had received adjuvanted quadrivalent vaccines (aIIV), with an effectiveness of 30% (95%CI: −6% to 54%). Among those aged 18–64, most of whom had received cell-based or recombinant vaccines, effectiveness was 47% [95%CI: 37–56%]. Among children aged 2–17, who mainly received live attenuated vaccines, effectiveness was 66% [95%CI: 53–76%] [9].

These findings are highly relevant, as the search for robust and effective vaccine platforms to improve influenza vaccination effectiveness in individuals aged 65 and older, as well as in immunocompromised patients, has become a global public health priority. However, in older adults, standard influenza vaccines have suboptimal immunogenicity and efficacy due to the phenomenon of immunosenescence and the relatively high prevalence of chronic conditions, including multimorbidity, in older adults [10,11]. Improved vaccines employ different strategies to enhance the immune response. To increase the magnitude and breadth of the immune response, currently, influenza vaccines with various adjuvants and increased antigen doses are in use, but their widespread implementation in practice remains limited [12]. In the Russian Federation, an inactivated subunit adjuvanted influenza vaccine was licensed over 25 years ago. It has been used within the framework of the National Immunization Schedule for individuals from 6 months of age through advanced old age, including pregnant women and individuals with compromised health conditions [13,14,15,16,17]. In connection with the continued circulation of coronavirus infection, there is a need to study changes in the picture of immunity formation in the population of the Russian Federation after influenza vaccination in the context of the impact of the past COVID-19 pandemic. Despite limited studies assessing the immunogenicity of influenza vaccines at the end of the pandemic, there are reports that in the 2022–2023 influenza season, inactivated influenza quadrivalent vaccines (IIV4) demonstrated strong immunogenicity, particularly in pregnant women and neonates. Studies showed a significant increase in antibody titers for all vaccine strains, with high seroprotection rates in both mothers and infants. The vaccines were also well-tolerated, with no severe adverse events reported [18]. So SARS-CoV-2 infection may alter the immune response to influenza vaccination. Interesting fact that patients who have had COVID-19 experience longer-lasting changes in the immune system, compared to those who have had other respiratory infections [19,20,21] and a constant fear about the outcome of the disease, aggravated the process of restoration of defects in various parts of the immune system [22].

Our study was conducted during the epidemiological period immediately following the COVID-19 pandemic, when SARS-CoV-2 circulation and the impact of previous infection (including potential long-term consequences, such as “long COVID”) remained significant factors for the general population. The aim of the study was a comparative assessment of the immunogenicity and safety of a quadrivalent inactivated subunit adjuvanted influenza vaccine in adults aged 18 to 85 during the final stage of the COVID-19 pandemic in the 2022–2023 season. All participants compared in the study had comparable rates of prior COVID-19 infection, weakening the assumption that post-COVID immune status is the key factor.

## 2. Materials and Methods

### 2.1. Study Design

**Figure 1:**
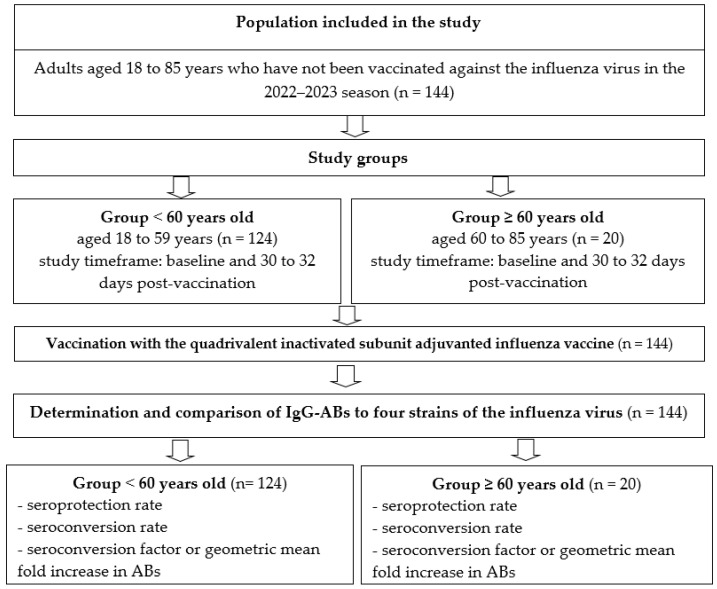
Study design.

A comparative cohort study was conducted involving 144 adults aged 18 to 85 who met the inclusion, non-inclusion, and exclusion criteria. No blinding was performed. The participants were divided into two age-based groups in accordance with the study design (Figure 1).

The study involved 144 influenza-vaccinated adults living in Moscow, Russia. They were divided into groups aged 18–59 years and aged 60–85 years. The target seroconversion rate was established at 60% (meeting predefined efficacy criteria), compared to an expected baseline rate of 30%, which required a minimum sample size of 20 participants in the 60–85 age group [23]. All participants were enrolled between October 2022 and April 2023.

Inclusion criteria: age between 18 and 85 years; no influenza immunization during the 2022–2023 season at the time of enrollment; provision of written informed consent by each volunteer.

Non-inclusion criteria: influenza vaccination in the 2022–2023 season; allergic reactions to chicken protein and vaccine components; allergic reactions to previously administered influenza vaccines; severe reaction (fever over 40 °C, swelling and redness at the injection site > 8 cm in diameter) or complications following previous influenza vaccination; allergic diseases; acute infectious or non-infectious diseases, or exacerbation of chronic conditions at the start of the study.

Exclusion criteria: non-compliance with the study protocol; withdrawal of consent or refusal to continue participation.

### 2.2. Study Site and Conditions

The study was conducted at the following institutions:

- Federal State Budgetary Scientific Institution “I.I. Mechnikov Research Institute of Vaccines and Sera”, Moscow, Russia;
- D. D. Pletnev City Clinical Hospital of the Health Department, Moscow, Russia;
- Federal State Autonomous Educational Institution of Higher Education I.M. Sechenov First Moscow State Medical University of the Ministry of Health of the Russian Federation (Sechenov University), Moscow, Russia.

The study was approved by the local ethics committee (extract from minutes No. 03-23 of the regular meeting of the Local Ethics Committee in Sechenov University dated 16 February 2023)

### 2.3. Collection of Data and Blood Samples

Demographic and clinical information of participants—including name, age, gender, medical history, and vaccine tolerability—was collected and recorded through examination and consultation over a period of 30–32 days following vaccination.

Venous blood samples were collected for serological analysis at baseline (Day 0, before vaccination) and again 30–32 days after vaccination. Following centrifugation, 3 mL of serum from each sample was transported to the “I.I. Mechnikov Research Institute of Vaccines and Sera” and stored at −50 °C.

### 2.4. Influenza Vaccine

The Grippol^®^ Quadrivalent vaccine (manufactured by NPO Petrovax Pharm LLC, Moscow region, Russia) is an influenza quadrivalent inactivated subunit adjuvanted vaccine manufactured in 2022, which was approved for use by the Ministry of Health of the Russian Federation. The product contains 5 µg of influenza virus antigens of type A (A/Victoria/2570/2019 (H1N1)pdm09-like virus, A/Darwin/9/2021 (H3N2)-like virus, type B (B/Phuket/3073/2013 (B/Yamagata lineage)-like virus, B/Austria/1359417/2021 (B/Victoria lineage)-like virus), and 500 µg of azoximer bromide (Polyoxidonium) per dose [24]. Before use, the product was stored in compliance with the temperature regime strictly according to the instruction. The vaccine in a dose of 0.5 mL was administered intramuscularly once into the deltoid muscle.

The development of the Grippol vaccine began in the 1990s, when Petrovax specialists, in collaboration with leading immunologists, began searching for effective adjuvants. The key idea was to create a polymer-subunit vaccine using the immunomodulator Polyoxidonium^®^ (azoximer bromide), manufactured by NPO Petrovax Pharm LLC, Moscow region, Russia, which would reduce the antigen dose while maintaining high efficacy and providing protection. The adjuvant Polyoxidonium^®^ is the only currently known water-soluble synthetic polymer adjuvant of the heterochain polyamine class and a copolymer of 1,4-ethylenepiperazine N-oxide and (N-carboxyethyl)-1,4-ethylenepiperazinium bromide (INN: Azoximer bromide) introduced into clinical practice. Polyoxidonium^®^ was developed and registered in Russia and has been used for over 25 years in the production of the Grippol family vaccines [25,26,27]. Pharmaceutical development and preclinical studies of the vaccine adjuvant were conducted over several years in the early 1990s. The drug was developed at the National Research Center—Institute of Immunology Federal Medical-Biological Agency [28]. As early as 1996, Polyoxidonium^®^ was approved for use in Russia (registration number 96/302/9, FS 42-3906-00). Since 2004, various forms of the drug have been approved for use in Slovakia, Georgia, Belarus, Kyrgyzstan, Ukraine, Kazakhstan, and Uzbekistan. The drug was the world’s first chemically pure high-molecular immunomodulator with complex action and as an adjuvant (at a dose of 500 mcg, determined to be optimal in experiments and clinical practice) for the production of influenza vaccines from the Grippol family [29].

Azoximer bromide is included only in influenza vaccines from the Grippol^®^ family (seasonal monovalent, trivalent and quadrivalent pandemic influenza vaccines). In 1999, Grippol^®^ was included in the National Immunization Schedule for epidemiological indications of the Russian Federation, and in 2006, it was added to the list of mandatory vaccines of the National Immunization Schedule.

### 2.5. Immunogenicity Assessment

Strain-specific antibody responses were assessed using a standard hemagglutination inhibition (HI) assay performed according to a standard operating procedure. To remove nonspecific hemagglutination inhibitors, sera were treated with receptor-destroying enzyme (RDE; Denka Seiken, Tokyo, Japan). Briefly, 50 µL of serum were mixed with 150 µL of RDE and incubated at 37 °C overnight (19 ± 1 h), followed by heat inactivation at 56 °C for 30 min and subsequent dilution to 1:10 in phosphate-buffered saline (PBS).

The HI assay was performed with 0.5% chicken red blood cells and 4 hemagglutination units of influenza antigens. Antigens for the HI assay were provided by the Smorodintsev Research Institute of Influenza (WHO National Influenza Centre of Russia, Saint Petersburg). The following antigen strains were used: A/Victoria/2570/2019 (H1N1)pdm09, A/Darwin/9/2021 (H3N2), B/Phuket/3073/2013, and B/Austria/1359417/2021.

As a reference for assessing the immunogenicity of the vaccine, the study used the vaccine efficacy criteria established for the general adult population by the Committee for Proprietary Medicinal Products (CPMP), protocol CPMP/BWP/214/96. These include:

- Seroprotection level—the percentage of vaccinated individuals who achieve a hemagglutination inhibition (HI) antibody titer of ≥1:40 by Day 21 after vaccination. This should be >70% for individuals under 60 years of age. For individuals aged 60 years and older, it should be >60%.
- Seroconversion rate or immunological activity of the vaccine—the proportion of vaccinated individuals whose HI antibody titer increases by more than fourfold compared to baseline levels among all seroprotected participants (≥4-fold titer increases). This should exceed 40% in individuals under 60 years of age. In individuals aged 60 years and older, it should exceed 30%.
- Seroconversion factor or geometric mean fold rise (GMFR)—the fold increase in the geometric mean titer (GMT) of HI antibodies by Day 21 compared to baseline. This should be >2.5 in individuals under 60 years of age. In individuals aged 60 years and older, it should be >2.0.

To be considered effective, a vaccine must meet at least one of the criteria listed above.

Serological studies to determine the levels of specific IgG-ABs were carried out by employees of the accredited Laboratory for Allergic Disease Prevention and Immunotherapy in Federal State Budgetary Scientific Institution “I. Mechnikov Research Institute of Vaccines and Sera” using certified equipment of the Shared Use Center at Federal State Budgetary Scientific Institution “I. Mechnikov Research Institute of Vaccines and Sera”, Moscow, Russian Federation.

### 2.6. Vaccine Safety Assessment

All participants were monitored for at least 30 min after vaccination to ensure safety and detect any acute adverse events following immunization (AEFIs). From Day 0 to Day 7, active follow-up was conducted via phone interviews. Participants could report any AEFIs to the investigators for up to 28 days after vaccination.

### 2.7. Statistical Analysis

Statistical analysis was performed in accordance with established guidelines for reporting clinical and observational vaccine study results [30].

The following vaccination efficacy indicators were calculated:

- Geometric Mean Fold Increase (GMFI) in IgG antibody titers, with descriptive statistics presented as the geometric mean and 95% confidence interval (CI). The measure of effect between groups was the Geometric Mean Fold Ratio (GMFR [95%CI]). Group comparisons were made using Student’s t-test (Welch’s test was applied if equal variance assumptions were violated) on log-transformed data.
- Seroprotection and seroconversion rates were reported as proportions with 95%CIs, calculated using the Clopper–Pearson method. The effect size was expressed as the Rate Difference (RD [95%CI]), and between-group comparisons were conducted using Barnard’s Exact Test.

The geometric mean titer (GMT) of IgG antibodies after vaccination was also analyzed. Descriptive statistics are presented as the geometric mean with a 95% confidence interval (CI). As a measure of the effect size between the groups, the Geometric Mean Ratio (GMR [95%CI]) was calculated.

Results are presented both without adjustment for baseline antibody levels (using Student’s *t*-test on log-transformed data) and with adjustment, applying ANCOVA that included baseline antibody levels and accounted for heteroscedasticity of residuals.

Differences were considered statistically significant at *p* ≤ 0.05. All calculations and graphical representations were performed using the R statistical environment (v.3.6, GNU GPL2 license).

## 3. Results

Baseline characteristics of compared groups (Table 1).

The groups were comparable in terms of gender composition (*p* < 0.16), vaccination season (*p* = 0.33), and the majority of examined comorbidities. However, in the group of participants aged over 60 years, there was a significantly higher frequency of previous myocardial infarction (*p* = 0.001) and lower limb vascular ischemia (*p* = 0.002).

The proportion of participants with a history of influenza vaccination during the 2021–2022 season was 59 individuals (48%) in the group 18–59 years and did not differ significantly from the group 60–85 years, which had 10 such cases (50%).

It is noteworthy that the proportion of respondents with a seropositive antibody level against the B/Victoria strain at baseline was higher in the age group over 60 years (*p* = 0.009) and the geometric mean fold increase (GMFI [95%CI]) in antibody levels against B/Victoria was also higher among older participants (*p* = 0.03).

### 3.1. Increase in Geometric Mean IgG Antibody Levels (Seroconversion Factor)

The first evaluated indicator of influenza vaccination effectiveness in adults was the Geometric Mean Fold Increase (GMFI) in IgG antibodies. The effect size between study groups was expressed as the Geometric Mean Fold Ratio (GMFR) (Table 2).

**Figure 2:**
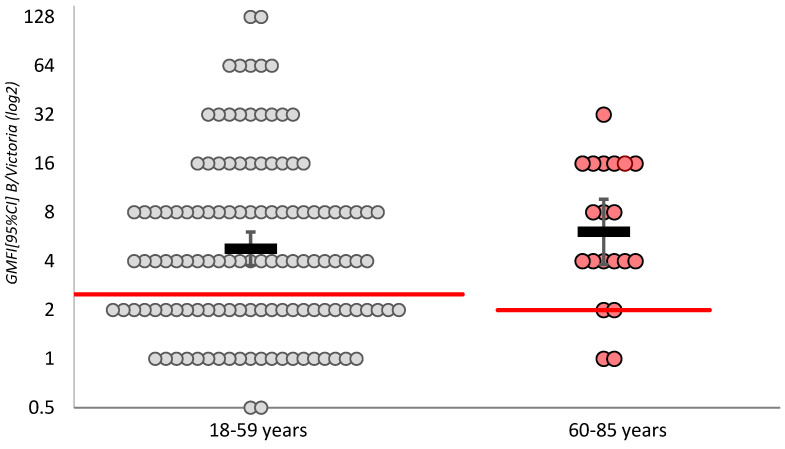

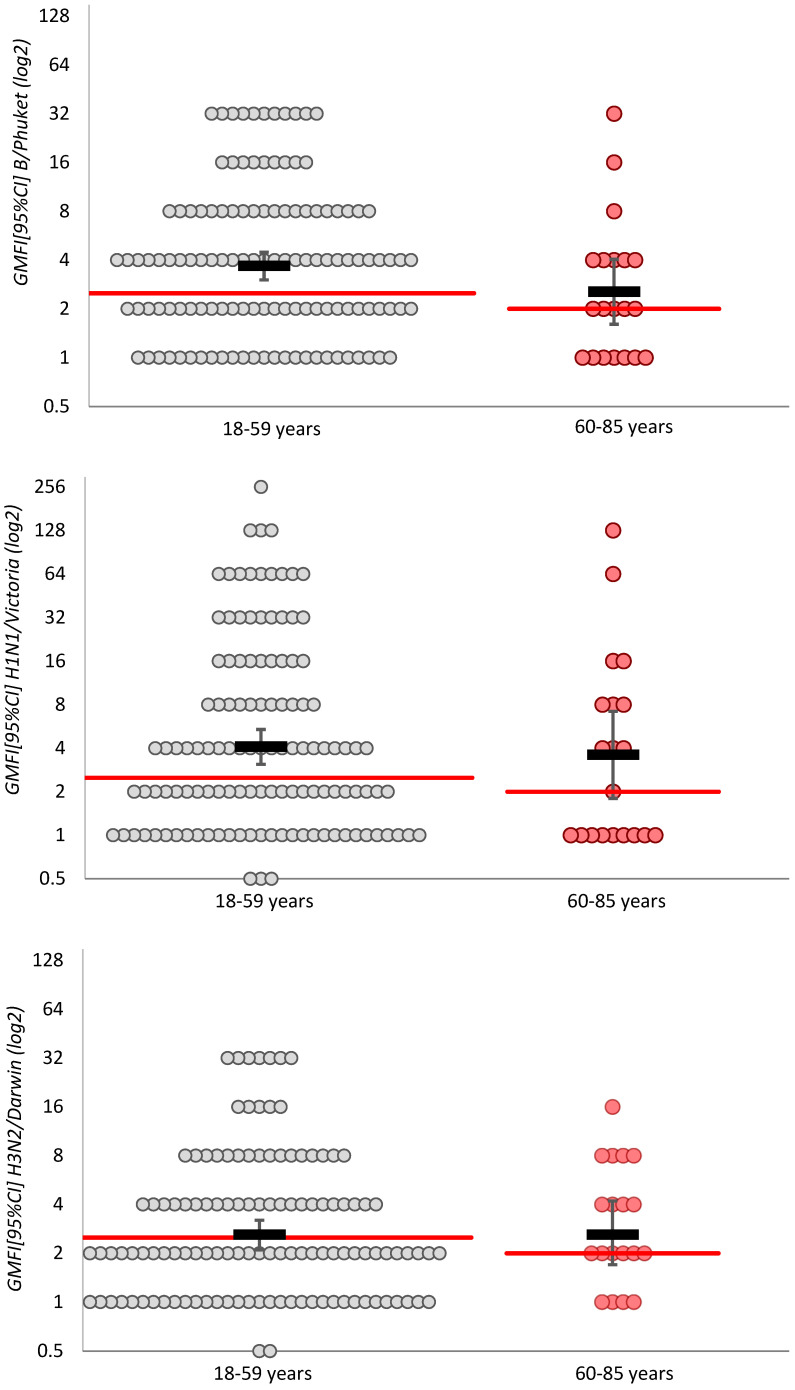
Geometric mean fold increase in the IgG antibodies level (GMFI [95%CI]) to influenza virus strains.

In both groups, the GMFI met the established effectiveness criteria, and no significant differences were found between the groups (Figure 2).

### 3.2. Seroprotection Rate

Next, the seroprotection rate (Seroprotection Rate [95%CI]) for influenza virus strains among vaccinated individuals was analyzed. The rate difference (RD [95%CI]) was calculated as the effect measure (Table 3).

**Figure 3:**
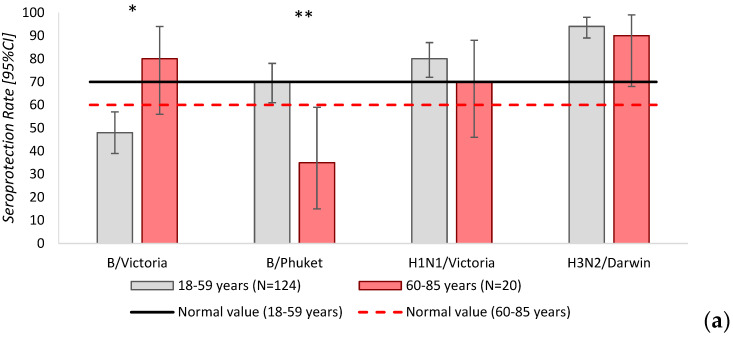

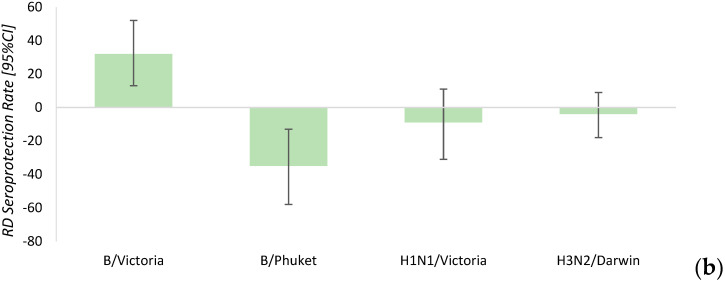
(**a**) Seroprotection rates for influenza virus strains among vaccinated individuals and 95% confidence intervals. *—statistically significant differences in seroprotection levels for B/Victoria between the study groups. **—statistically significant differences in seroprotection levels for B/Phuket/3073/13 between the study groups. (**b**) Differences in seroprotection rates for influenza virus strains between study groups.

In the group of patients 18–59 years, the seroprotection rate for B/Victoria was below the threshold of effectiveness. In the group of respondents aged 60–85 years, the seroprotection rate for B/Phuket/3073/13 was below the threshold of effectiveness. Statistically significant differences in seroprotection levels for B/Victoria and B/Phuket/3073/13 were identified between the study groups (Figure 3).

The seroconversion rate [95%CI] for influenza virus strains among vaccinated individuals and the difference between study groups (Rate Difference—RD [95%CI]) are presented in Table 4.

**Figure 4:**
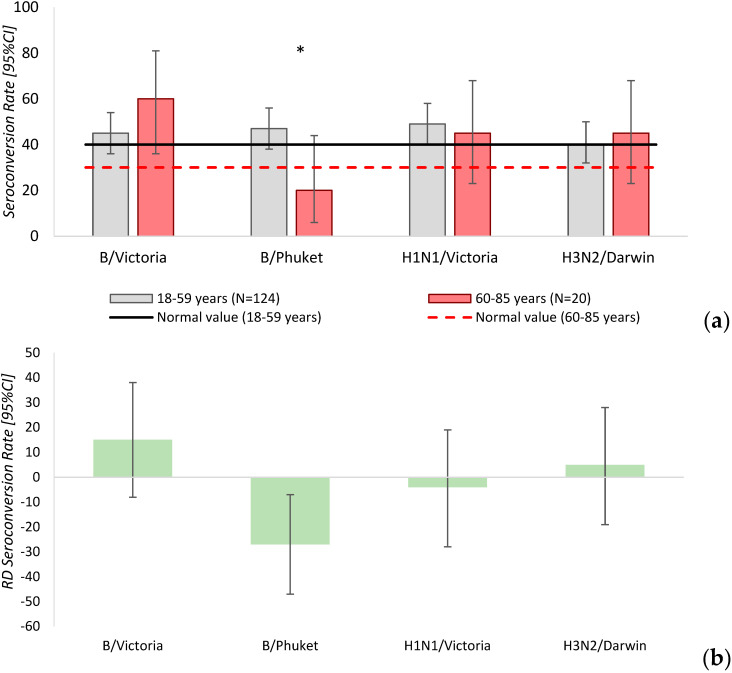
(**a**) Seroconversion rates to influenza virus strains in vaccinated individuals with 95% confidence intervals. *—statistically significant differences between the study groups. (**b**) Difference in seroconversion rates to influenza virus strains between study groups.

In the group of participants 18–59 years, the seroconversion rate for all influenza strains met the established efficacy criteria. In the group of patients 60–85 years the seroconversion rate for the B/Phuket/3073/13 influenza strain was below the efficacy threshold. A difference in seroconversion rate for the B/Phuket/3073/13 strain was observed between the study groups (RD = −27 [95%CI: −7–−47], *p* = 0.05) (Figure 4).

The overall results for meeting vaccination efficacy criteria are summarized in Table 5.

### 3.3. Geometric Mean IgG Antibody Titer (Seroconversion Factor), with and Without Baseline Adjustment

Next, we examine the geometric mean IgG antibody titer (Geometric Mean Titer—GMT) after vaccination in adult groups. Results are presented both without baseline adjustment and with baseline adjustment (Baseline-corrected) in Table 6. In both cases, the effect size was calculated as the Geometric Mean Ratio (GMR [95%CI]). For the baseline-adjusted calculation, GMR was estimated using an ANCOVA model with correction for heteroscedasticity of residuals.

Statistically significant differences in post-vaccination geometric mean IgG antibody titers between study groups were found for the B/Victoria strain: 67.3 [95%CI: 42.1–108] in the group 60–85 years versus 30.1 [95%CI: 23.9–37.9] in the group 18–59 years (*p* = 0.003). However, the analysis adjusted for baseline titers showed that these differences were due to initially higher titers against B/Victoria in the older group (GMR = 1.37 [95%CI: 0.88–2.15], *p* = 0.15), which is also supported by the lack of difference in seroconversion rates for this strain.

**Figure 5:**
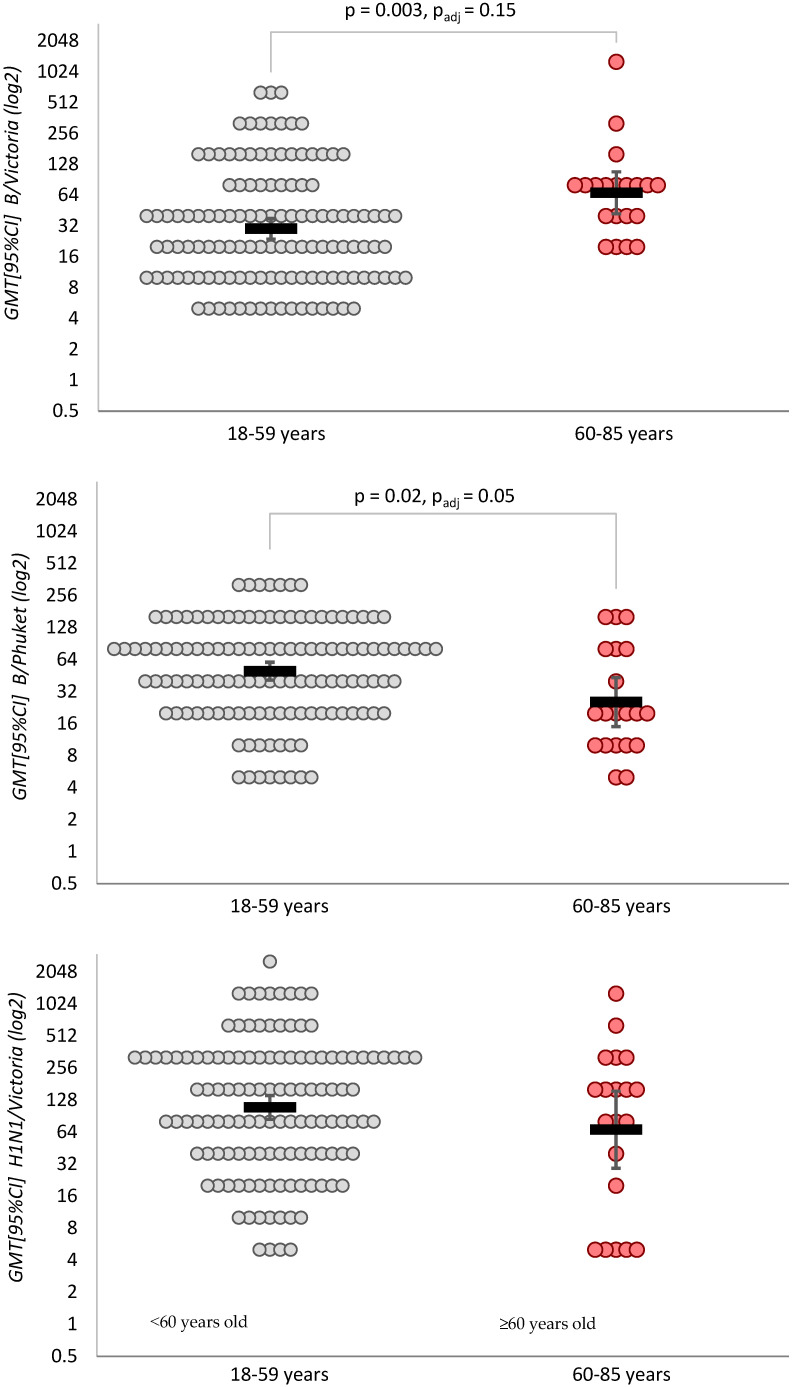

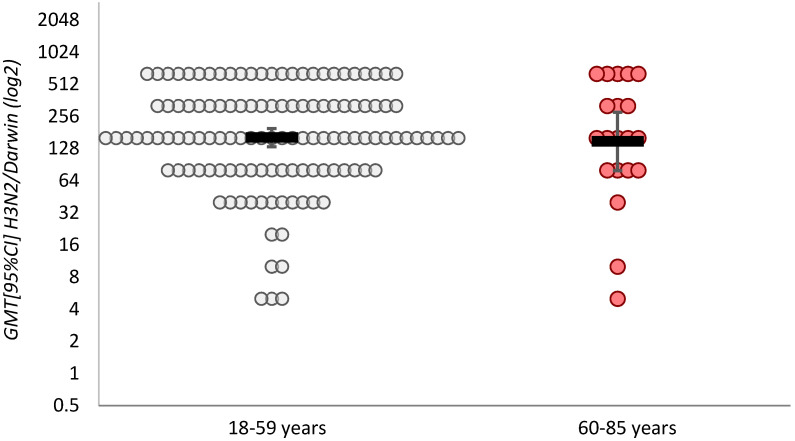
Geometric mean IgG antibody titers to influenza virus strains in adults after vaccination. *p* = Student’s *t*-test (for equal variances) or Welch’s test (for unequal variances) for log-transformed data; p_adj_ = ANCOVA with inclusion of baseline antibodies level and correction for heteroscedasticity of residuals.

In contrast, differences in geometric mean IgG titers to the B/Phuket/3073/13 strain were confirmed both by the unadjusted analysis (25.5 [95%CI: 15–43.3] in the group 60–85 years vs. 49.7 [95%CI: 41.1–60.2] in the group 18–59 years, GMR [95%CI] = 0.51 [95%CI: 0.29–0.89], *p* = 0.02) and by the adjusted analysis (GMR [95%CI] = 0.74 [95%CI: 0.54–1.00], *p* = 0.05) (Figure 5).

### 3.4. Vaccination Safety

In the group 18–59 years of age, local post-vaccination reactions manifested as pain, redness, and swelling at the injection site (Table 7). Systemic reactions included general malaise, rhinitis, myalgia, fever (not exceeding 38.0 °C), fatigue, and drowsiness. Overall, local and systemic reactions occurred within the first few days after vaccination. No serious adverse events or reactions requiring medical intervention occurred during the one-month follow-up period. All reactions resolved spontaneously within 1–3 days post-vaccination.

Among vaccinated individuals 60–85 years old, no unusual reactions requiring medical attention were reported within the one-month follow-up either. All reactions resolved on their own within 1–3 days after vaccination, either without medication or with the use of antipyretics.

No significant differences in the frequency or intensity of post-vaccination reactions were observed between the two adult groups receiving the quadrivalent inactivated subunit adjuvanted influenza vaccine.

## 4. Discussion

The initiation of the present study on post-vaccination immunity to influenza viruses was prompted by previously described evidence suggesting the existence of multisystem humoral and immune responses combined with numerous physical, neurological, and psychiatric symptoms after recovery from COVID-19 infection, characterizing the pathogenesis of residual long COVID syndrome [31,32,33]. Symptoms of post-COVID syndrome were observed even in patients with prior mild or moderate COVID-19, who reported persistence of symptoms for more than 4 months after infection [34,35]. It was hypothesized that these factors could affect both the specific immune response and the tolerability of influenza vaccination.

The rise in influenza incidence amid declining SARS-CoV-2 infections in 2022–2023 was a positive factor encouraging voluntary participation in our study on the immunogenicity of the quadrivalent inactivated subunit adjuvanted influenza vaccine in adults under and over 60 years of age.

Comparative aspects of evaluating post-vaccination immunity among adults aged 18–59 years and those aged 60–85 years demonstrated comparable results for the A/Victoria/2570/2019 (H1N1)pdm09-like virus and A/Darwin/9/2021 (H3N2)-like virus strains, meeting accepted immunogenicity criteria. For the A(H1N1) strain, in the 18–59 years/60–85 years age groups, the seroprotection rate (required ≥70%/≥60%) was 80% [95%CI: 72–87]%/70% [95%CI: 46–88]% (*p* = 0.38), the seroconversion rate (required ≥40%/≥30%) was 49% [95%CI: 40–58]%/45% [95%CI: 23–68]% (*p* = 0.53), and the geometric mean fold increase in IgG antibody titers (seroconversion factor, required ≥2.5/≥2.0) was 4.16 [95%CI: 3.17–5.46]/3.61 [95%CI: 1.79–7.26] (*p* = 0.69).

A difference in seroprotection levels between study groups was identified for the B/Austria/1359417/2021 strain, with levels below the ≥70% threshold in the 18–59 age years group compared to the 60–85 years group (*p* = 0.01). The accepted seroprotection threshold for this strain is ≥60%.

Conversely, for the B/Phuket/3073/13 strain, the seroprotection level was below the threshold in the 60–85 years group, compared to the 18–59 age years group (*p* = 0.007).

A similar pattern was observed when assessing geometric mean IgG antibody titers for influenza B strains in vaccinated individuals. For the B/Austria/1359417/2021 strain, titers were lower in the 18–59 years group compared to the 60–85 age years group (*p* = 0.003). In contrast, for the B/Phuket/3073/13 strain, titers were higher in the 18–59 age years group compared to the 60–85 age years group (*p* = 0.02).

Analysis adjusted for baseline IgG antibody titers showed that these differences were explained by initially higher antibody titers to the B/Austria/1359417/2021 strain in the 60–85 age years group (*p* = 0.15), which is also supported by the absence of differences in seroconversion rates for this strain.

Seroconversion rates for the B/Austria/1359417/2021 strain in the observed groups met the immunogenicity criteria, whereas for the B/Phuket/3073/13 strain in the 60–85 age years group, the required threshold of ≥30% was not reached. This contrasted with the comparable indicator in participants 18–59 years old, which met the ≥40% threshold (*p* = 0.05).

Although we observed a reduced seroprotection and seroconversion against B/Yamagata in the older age group, the small number of cases limited the statistical power. These findings preclude definitive conclusions regarding vaccination efficacy for this strain. The results should be considered preliminary and require confirmation in larger-scale studies.

Differences in influenza vaccine efficacy across age groups may also be explained by potential imprinting effects (human immunological memory to previously encountered influenza viruses). Molecular-genetic studies of influenza B virus strains circulating from 2020 to 2023 have shown their affiliation with the B/Victoria-like lineage, clade V1A.3a.2 [2,36,37]. Recent epidemic strains contained amino acid substitutions that caused a phenotypic “reversion” to viruses with antigenic properties similar to those circulating over 50 years ago [38,39].

As mentioned earlier, modern quadrivalent influenza vaccines include two influenza A viruses (A(H1N1)pdm09 and A(H3N2)) and two influenza B viruses from different evolutionary lineages (B/Victoria-like and B/Yamagata-like), which diverged in the 1970s and have since been co-circulating [40,41]. Since March 2020, surveillance worldwide has reported an absence of active circulation of B/Yamagata-like viruses. It is possible that the lack of natural boosting, along with a weaker immune response in older adults, accounts for the failure to meet some immunogenicity criteria in this group. Nevertheless, the B/Phuket/3073/13 strain (B/Yamagata lineage-like virus) in the over-60 group demonstrated a geometric mean fold increase in IgG antibody titers of 2.55 [95%CI: 1.61–4.05], meeting the immunogenicity criterion of ≥2.0.

Based on the accepted 3 criteria for assessing the immunogenicity of vaccines developed for a standard adult, established by the CPMP—protocol CPMP/BWP/214/96, for a vaccine to be considered effective, it must meet the specified requirements for at least one of the specified indicators. In individuals 60–85 years of age, its immunogenicity has been proven by one of the criteria as an increase in the geometric mean level of IgG antibodies (GMFI [95%CI]) (seroconversion factor) to the B/Yamagata strain of the influenza virus (Table 2), that is, for this cohort, IgG antibodies are synthesized and with repeated administration of vaccines (in subsequent seasons), higher levels of specific IgG antibodies to the specified strain can be observed.

Literature data indicate that among the four types of viruses included in seasonal influenza vaccines—A(H1N1), A(H3N2), B/Victoria, and B/Yamagata—the influenza A(H3N2) virus is most commonly associated with reduced vaccine effectiveness. Moreover, waning immunity during the influenza season has been reported more frequently for A(H3N2) than for other virus types [42,43,44,45].

In our study, regardless of age, a significant post-vaccination increase in specific antibodies to the A(H1N1) and A(H3N2) strains was observed. This finding aligns with data from a systematic literature review presenting studies on the absolute and relative immunogenicity, effectiveness, and safety of the MF59-adjuvanted trivalent influenza vaccine (aTIV) and the MF59-adjuvanted quadrivalent influenza vaccine (aQIV) in non-elderly adults (<65 years) [46].

It was established that vaccines containing adjuvant (aTIV/aQIV) demonstrated greater immunogenicity compared to non-adjuvanted vaccines for the vaccine-like strains: absolute differences in seroconversion rates were 8.8% [95%CI: 3.7– 14.0]%, 13.1% [95%CI: 6.7–19.6]%, and 11.7% [95%CI: 7.2–16.2]% for A(H1N1), A(H3N2), and B strains, respectively. A significantly enhanced immune response following aTIV administration compared to standard non-adjuvanted trivalent influenza vaccines (TIV) in elderly individuals has also been described by other authors [47].

It is important to emphasize that vaccine safety is a crucial parameter reflecting vaccine quality, especially when new technological constructs, including conjugates and adjuvants, are used.

Analysis of the safety and immunogenicity profile of the influenza subunit vaccine with the adjuvant Azoximer bromide in the analysis of 30 studies from 1993 to 2016 containing data on 11,736 participants using the adjuvanted inactivated influenza vaccine with Azoximer bromide, to compare the reactogenicity and immunogenicity of the vaccine with non-adjuvanted vaccines, it was shown that the Grippol^®^ family of vaccines effectively induce an immune response; shows lower local reactogenicity compared to split and subunit non-adjuvanted vaccines; have a favorable safety profile and immunogenicity of vaccines, along with a reduced amount of antigen per dose and the gentle effect of Azoximer bromide, make Grippol^®^ vaccines good candidates for use not only during a pandemic, but also in general for vaccination against seasonal influenza [16].

Our analysis of studies on the immunogenicity of the original trivalent and later quadrivalent subunit adjuvanted influenza vaccine (based on azoximer bromide)—Grippol Plus and Grippol Quadrivalent—published before 2020 in adults (including people aged 18–60 years and over 60 years) allows us to draw the following conclusions regarding seroprotection and seroconversion rates (Table 8) [48,49,50]:

- Trends for A(H1N1) and A(H3N2) strains: seroprotection and seroconversion rates for influenza A strains in our 2022–2023 study in both age groups are consistent with or even exceed typical ranges observed pre-COVID for this vaccine type (seroprotection > 70–80% for adults < 60 years and >60–70% for individuals ≥ 60 years; seroconversion > 40% and >30%, respectively).
- Trends for influenza B strains (Victoria and Yamagata lineages): A more variable pattern is observed, consistent with the known characteristics of the immune response to the B virus.

**Table 8:** Immunogenicity of the trivalent (Grippol Plus) and quadrivalent (Grippol Quadrivalent) subunit adjuvanted influenza vaccine in adults 18–60 years/≥60 years.

Immunogenicity Parameter of the Vaccine	Standards of the CPMP *	Results, Influenza Season, Age							
		Grippol Family						Influvac	Vaxigrip
		Plus			Quadrivalent				
		2012–2013	2016–2017	2016–2017	2016–2017	2022–2023	2022–2023	2016–2017	2016–2017
		≥60	18–55	18–60	18–60	18–59	60–85	18–55	18–55
A/H1N1									
Seroconversion factor	≥2.5/≥2.0	6.5	7.20	4.60	4.90	4.16	3.61	7.57	8.06
Seroconversion rate	≥40%/≥30%	67.8%	93.2%	62.4%	65.8%	49%	45%	94.6%	94.4%
Seroprotection level	≥70%/≥60%	78.8%	95.0%			80%	70%	95.0%	96.0%
A/H3N2									
Seroconversion factor	≥2.5/≥2.0	3.4	3.78	5.17	5.31	2.6	2.64	4.59	4.32
Seroconversion rate	≥40%/≥30%	49.2%	67.4%	62.9%	69.3%	40%	45%	77.8%	92.5% *
Seroprotection level	≥70%/≥60%	67.4%	90.9%			94%	90%	90.0%	96.0%
B/Victoria									
Seroconversion factor	≥2.5/≥2.0	2.6	2.70	4.79	4.77	4.7	6.06	2.50	3.27
Seroconversion rate	≥40%/≥30%	39.0%	71.4%	62.9%	67.8%	45%	60%	90.0%	93.8%
Seroprotection level	≥70%/≥60%	79.7%	99.0%			48%	80%	100.0%	100.0%
B/Yamagata									
Seroconversion factor	≥2.5/≥2.0				5.47	3.68	2.55		
Seroconversion rate	≥40%/≥30%				65.3%	47%	20%		
Seroprotection level	≥70%/≥60%					70%	35%		

*—The requirements of the international standards CPMP EMEA, CPM/EWP/1045/01 for adults 18–60 years/≥60 years: for individuals over 60 years of age, an influenza vaccine is considered immunogenic if it meets at least one of the following criteria: seroprotection rate ≥ 60%, seroconversion rate ≥ 30%, antibody titer increase ≥ 2.0.

Our vaccination conducted at the end of the COVID-19 pandemic in the 2022–2023 season among adults under and over 60 years old was not accompanied by the occurrence of unusual adverse events. The frequency of mild local post-vaccination reactions in the 18–59 years group (30 individuals, 24.2%) was similar to that in the 60–85 age years group (4 individuals, 20%). Systemic reactions such as general malaise, rhinitis, myalgia, fever not exceeding 38 °C, fatigue, and somnolence of mild severity were reported in 14 (11.3%) vaccinated individuals under 60 years and were comparable to 3 (15%) cases in the 60–85 age years group. These reactions did not differ from those described in the vaccine’s product information during the pre-pandemic period.

The aTIV/aQIV influenza vaccines used with other adjuvants were also found to be safe and well tolerated, although the incidence of post-vaccination reactions was higher than with adjuvant-free vaccine products. Serious adverse events were rare and no difference (risk ratio 1.02 [95%CI: 0.64–1.63]) was found between aTIV/aQIV and non-adjuvanted vaccines [46].

To enhance protection against influenza in older adults, the WHO, the Advisory Committee on Immunization Practices (ACIP), and other agencies recommend enhanced influenza vaccines for this population [51,52]. Recommended licensed vaccines include MF59-adjuvanted inactivated influenza vaccine (aIIV; Fluad or Fluad Quadrivalent; CSL Seqirus Ltd. Parkville, Australia), high-dose inactivated influenza vaccine (HD-IIV; Fluzone High Dose and Fluzone High Dose Quadrivalent, Sanofi, Paris, France), and (higher than standard dose) quadrivalent recombinant influenza vaccine (RIV4; FluBlok Quadrivalent, Sanofi, Paris, France). Higher dose vaccines contain either 60 μg HA per strain (HD-IIV) or 45 μg HA per strain (RIV4), which increases the magnitude of the immune response compared to standard influenza vaccines containing 15 μg HA per strain [53]. The aIIV vaccine contains a standard dose of antigen (i.e., 15 μg HA per strain) enhanced with the MF59 adjuvant, an oil-in-water emulsion based on squalene, which increases not only antibody synthesis but also the breadth of the immune response by enhancing the production of cross-reactive antibodies [54]. This feature is particularly important during seasons when circulating influenza viruses do not match the vaccine strains.

We managed to find works that assessed the formation of post-vaccination immunity after the introduction of vaccines of national vaccination calendars conducted during the pandemic. As is known, the pandemic situation reduced the level of vaccination coverage across the planet, and then the WHO directive was issued on complex (from the moment of use of 3–4 vaccines) catch-up vaccination (when the use of vaccines was allowed at the request of parents at any time upon appeal, without observing the intervals of four weeks between vaccinations.

During the COVID-19 pandemic, studies on influenza vaccines focused on their immunogenicity and safety, particularly when co-administered with COVID-19 vaccines. Research explored whether co-administration impacted the immune response to either vaccine and whether a combined influenza and COVID-19 vaccine (mRNA-1083) could offer a more efficient approach [55]. Several key findings emerged: Co-administration is generally safe and effective; study on mRNA-1083, a combined influenza and COVID-19 vaccine, demonstrated that it met non-inferiority criteria and induced higher immune responses against all four influenza strains (in those aged 50–64) and the three clinically relevant strains (in those aged 65+) compared to standard care vaccines. It also showed good immunogenicity against SARS-CoV-2; a study found that COVID-19 vaccination could enhance the immunogenicity of influenza vaccines in older adults, potentially boosting influenza virus vaccine-induced serum HAI activity while maintaining COVID-19 protective immunity.

At the same time, there are data showing advantages in the formation of post-vaccination antibodies against SARS-CoV-2 with separate administration of vaccines against COVID-19 and influenza, while the level of antibodies to the influenza virus did not depend on the vaccination method [56].

The relationship between the national calendar vaccines received before the start of the pandemic and the concept of trained immunity is also discussed [57].

A study conducted in China during the pandemic in children showed that the geometric mean titer of antibodies against DTaP and MMR in the simultaneous vaccination group was non-inferior to those in the DTaP alone and MMR alone group. Reporting rates of local and systemic adverse reactions were similar between groups and no serious adverse events were reported throughout the clinical study period [58]. However, the source does not indicate the timing of the study, or whether the decrease in antibody levels is associated with the mechanism of action of combination vaccines or as a consequence of COVID-19.

In our study, we demonstrated the immunological efficacy of a quadrivalent inactivated subunit influenza vaccine containing the adjuvant. The vaccine design is based on a unique technology that complexes highly purified protective influenza virus antigens with a polymeric, water-soluble high-molecular-weight immunoadjuvant—Azoximer bromide—at a dose of 500 µg per 0.5 mL vaccine dose. This formulation allows for a threefold reduction in the hemagglutinin (HA) content per strain (down to 5 µg), compared to the 15 µg HA found in non-adjuvanted subunit vaccines. On one hand, this represents an antigen-sparing technology that enables the production of a larger number of vaccine doses; on the other hand, it is antigen-sparing for the human body, reducing the incidence of adverse reactions, which is critically important when performing simultaneous vaccination against multiple infections [24]. The practical application of this production technology in Russia and Commonwealth of Independent Countries has been ongoing for over 20 years.

Clinical trials and observational studies have generally shown that, compared to standard influenza vaccines, improved vaccine technologies that activate molecular and cellular mechanisms enhance vaccine immunogenicity. This enhancement translates into better protection against influenza infection, as well as reductions in physician visits, hospitalizations, and complications among vaccinated healthy individuals and those with various health conditions [16,59,60,61,62].

Other studies involving direct comparisons with non-adjuvanted vaccines have also demonstrated that administration of adjuvanted vaccines is associated with increased immunogenicity and a broader immune response against drifted influenza virus strains [63,64].

A limitation of the study is the lack of comparison of Polyoxidonium with other adjuvants. To date, we are not aware of any comparative studies of Azoximer bromide with other adjuvants, although interesting insights could have been gained from such research.

The production technology of the tetravalent inactivated subunit adjuvant vaccine against influenza is quite straightforward: a polymeric water-soluble high-molecular immunoadjuvant, Azoximer bromide, is added to purified influenza virus antigens at a dosage of 500 μg per vaccine dose (0.5 mL). It is important to note that Azoximer bromide is a well-known immunomodulator, Polyoxidonium^®^, which belongs to the class of synthetic polyelectrolytes [65]. This compound is an N-oxidized derivative of polyethylenepiperosine with a high molecular weight and is utilized to treat and prevent diseases associated with immune system disorders. Polyoxidonium^®^ was developed in the Russian Federation. According to toxicity classification, Polyoxidonium^®^ falls into class 5, indicating that it is practically non-toxic. The high safety profile of Polyoxidonium^®^ is further supported by preclinical studies, which demonstrate that the drug does not exhibit pyrogenic, irritating, toxic, allergenic, mutagenic, embryotoxic, teratogenic, or carcinogenic properties even at doses 50 times higher than the therapeutic dose. The inclusion of Polyoxidonium^®^ in the Grippol^®^ vaccine has allowed for a reduction in the amount of antigen required (5 μg of hemagglutinin from serotypes A/H1N1, A/H3N2, and B).

The study’s primary focus on humoral responses represents another limitation, particularly in the context of elderly populations where cellular immune responses may play a crucial role. While humoral immunity provides valuable insights into vaccine efficacy, it does not fully capture the complexities of the immune response, especially in older adults who often exhibit diminished humoral responses. Acknowledging this limitation is essential for accurately interpreting the overall immunogenic profile of the vaccine.

The exploration of cellular immunity could significantly enrich our understanding of vaccine-induced protection, as it encompasses various mechanisms that contribute to long-term immunity and resistance to infections. Previous research has highlighted the importance of cellular immune responses, particularly in studies involving adjuvanted influenza vaccines. For instance, findings from studies on patients with bronchial asthma and COPD demonstrated correlations between clinical outcomes and inflammation markers, showcasing how cellular mechanisms can influence vaccine effectiveness over time [62]. Therefore, incorporating an assessment of cellular immune responses would not only address this weakness but also provide a more comprehensive view of the vaccine’s immunogenicity, ultimately leading to better-informed strategies for protecting vulnerable populations, such as the elderly.

## 5. Conclusions

Adjuvants remain a critical strategy for enhancing the effectiveness of both traditional and next-generation vaccines, and the selection of adjuvanted vaccines offers clear practical advantages in ensuring effective protection against influenza. The studies evaluating the specific immune response to a quadrivalent inactivated subunit adjuvanted influenza vaccine during the post-COVID-19 pandemic period in the 2022–2023 season among adults younger and older than 60 years demonstrated that the vaccine meets established immunogenicity and safety criteria in respondents aged 18 to 85 years despite post-COVID changes in immunity.

## Figures and Tables

**Table 1:** Baseline characteristics of compared groups.

Indicators	Total	18–59 Years	60–85 Years	*p* *
**Medical history**				
M/F	65/79	53/71	12/8	*p* = 0.16
Myocardial infarction	8 (6%)	3 (2%)	5 (25%)	*p* = 0.001
Type 2A, 2B, and 3 chronic heart failure	7 (5%)	4 (3%)	3 (15%)	*p* = 0.06
Lower limb ischemia	3 (2%)	0 (0%)	3 (15%)	*p* = 0.002
Aortic aneurysm	1 (1%)	1 (1%)	0 (0%)	*p* = 1.0
COPD	4 (3%)	3 (2%)	1 (5%)	*p* = 0.45
Connective tissue disorders	1 (1%)	1 (1%)	0 (0%)	*p* = 1.00
Gastric ulcer	7 (5%)	4 (3%)	3 (15%)	*p* = 0.07
Chronic hepatitis B	5 (3%)	5 (4%)	0 (0%)	*p* = 1.00
Diabetes mellitus	10 (7%)	7 (6%)	3 (15%)	*p* = 0.10
Vaccination in the previous season	69 (48%)	59 (48%)	10 (50%)	*p* = 0.84
Contracted COVID-19	82 (57%)	68 (54%)	14 (70%)	*p* = 0.23
Experienced severe COVID-19	13 (9%)	9 (7%)	4 (20%)	*p* = 0.08
**Vaccination season**				
Fall 2022	67 (47%)	56 (45%)	11 (55%)	*p* = 0.33
Winter 2023	68 (47%)	61 (49%)	7 (35%)	
Spring 2023	9 (6%)	7 (6%)	2 (10%)	
**Proportion of initially seropositive respondents—*n* (%) [95%CI]**				
B/Austria/1359417/2021 (B/Victoria lineage)-like virus	12 (8%) [4–14]	7 (6%) [2–11]	5 (25%) [9–49]	*p* = 0.01
B/Phuket/3073/13 (B/Yamagata lineage)-like virus	31 (22%) [15–29]	28 (23%) [16–31]	3 (15%) [3–38]	*p* = 0.57
A/Victoria/2570/2019 (H1N1)pdm09-like virus	61 (42%) [34–51]	53 (43%) [34–52]	8 (40%) [19–64]	*p* = 0.82
A/Darwin/9/2021 (H3N2)-like virus	104 (72%) [64–79]	91 (73%) [65–81]	13 (65%) [41–85]	*p* = 0.44
**Baseline GMT [95%CI]**				
B/Austria/1359417/2021 (B/Victoria lineage)-like virus	6.9 [6.12–7.79]	6.39 [5.71–7.16]	11.1 [6.75–18.2]	*p* = 0.03
B/Phuket/3073/13 (B/Yamagata lineage)-like virus	13 [11–15.3]	13.5 [11.4–16.1]	10 [6.04–16.6]	*p* = 0.25
A/Victoria/2570/2019 (H1N1)pdm09-like virus	25 [19.7–31.5]	26.2 [20.4–33.5]	18.7 [8.81–39.5]	*p* = 0.38
A/Darwin/9/2021 (H3N2)-like virus	61.4 [49.5–76.2]	62.2 [49.6–78]	56.6 [27.4–117]	*p* = 0.79

Notes: * For quantitative indicators, comparisons were performed using either Student’s *t*-test (for equal variances) or Welch’s test (for unequal variances) on log-transformed data; for categorical indicators, Barnard’s Exact Test was used.

**Table 2:** Geometric mean fold increase in IgG-ABs (GMFI [95%CI]) to influenza virus strains and the geometric mean fold ratio (GMFR [95%CI]) in the study groups.

Indicators	GMFI [95%CI]		GMFR [95%CI]	*p* *
	18–59 Years	60–85 Years		
Normal range	≥2.5	≥2.0	-	-
B/Austria/1359417/2021 (B/Victoria lineage)-like virus	4.7 [3.77–5.87]	6.06 [3.81–9.64]	1.28 [0.77–2.13]	*p* = 0.31
B/Phuket/3073/13 (B/Yamagata lineage)-like virus	3.68 [3.02–4.48]	2.55 [1.61–4.05]	0.69 [0.42–1.14]	*p* = 0.14
A/Victoria/2570/2019 (H1N1)pdm09-like virus	4.16 [3.17–5.46]	3.61 [1.79–7.26]	0.86 [0.41–1.82]	*p* = 0.69
A/Darwin/9/2021 (H3N2)-like virus	2.6 [2.11–3.2]	2.64 [1.66–4.2]	1.01 [0.61–1.68]	*p* = 0.95

Notes: * Student’s *t*-test (for equal variances) or Welch’s test (for unequal variances) for log-transformed data was applied.

**Table 3:** Seroprotection rate [95%CI] for influenza virus strains among vaccinated individuals and the difference between study groups (RD [95%CI]).

Parameter	Seroprotection Rate [95%CI]		RD [95%CI]	*p* *
	18–59 years	60–85 years		
	*n* = 124	*n* = 20		
Normal range heart failure	≥70%	≥60%	-	-
B/Austria/1359417/2021 (B/Victoria lineage)-like virus	59 (48%) [39–57]	16 (80%) [56–94]	32% [13–52]	*p* = 0.01
B/Phuket/3073/13 (B/Yamagata lineage)-like virus	87 (70%) [61–78]	7 (35%) [15–59]	−35% [−13–58]	*p* = 0.007
A/Victoria/2570/2019 (H1N1)pdm09-like virus	99 (80%) [72–87]	14 (70%) [46–88]	−9 [−31–11]	*p* = 0.38
A/Darwin/9/2021 (H3N2)-like virus	117 (94%) [89–98]	18 (90%) [68–99]	−4 [−18–9]	*p* = 0.55

Notes: * Barnard’s Exact Test was applied.

**Table 4:** Seroconversion rate [95%CI] for influenza virus strains among vaccinated individuals and its difference between study groups (RD [95%CI]).

Parameter	Seroconversion Rate [95%CI]		RD [95%CI]	*p* *
	18–59 years	60–85 years		
Normal range heart failure	≥40%	≥30%	-	-
B/Austria/1359417/2021 (B/Victoria lineage)-like virus	56 (45%) [36–54]	12 (60%) [36–81]	15 [−8–38]	*p* = 0.24
B/Phuket/3073/13 (B/Yamagata lineage)-like virus	58 (47%) [38–56]	4 (20%) [6–44]	−27 [−7–−47]	*p* = 0.05
A/Victoria/2570/2019 (H1N1)pdm09-like virus	61 (49%) [40–58]	9 (45%) [23–68]	−4 [−28–19]	*p* = 0.53
A/Darwin/9/2021 (H3N2)-like virus	50 (40%) [32–50]	9 (45%) [23–68]	5 [−19–28]	*p* = 0.92

Notes: * Barnard’s Exact Test was applied.

**Table 5:** Composite outcomes for vaccination efficacy criteria.

Virus Strains	GMFI	Seroprotection Rate	Seroconversion Rate
**Age 18–59 years**			
B/Austria/1359417/2021 (B/Victoria lineage)-like virus	Yes	No	Yes
B/Phuket/3073/13 (B/Yamagata lineage)-like virus	Yes	Yes	Yes
A/Victoria/2570/2019 (H1N1)pdm09-like virus	Yes	Yes	Yes
A/Darwin/9/2021 (H3N2)-like virus	Yes	Yes	Yes
**Age 60–85 years**			
B/Austria/1359417/2021 (B/Victoria lineage)-like virus	Yes	Yes	Yes
B/Phuket/3073/1 (B/Yamagata lineage)-like virus	Yes	No	No
A/Victoria/2570/2019 (H1N1)pdm09-like virus	Yes	Yes	Yes
A/Darwin/9/2021 (H3N2)-like virus	Yes	Yes	Yes

**Table 6:** Geometric mean titer of IgG antibodies [95%] to influenza virus strains in vaccinated individuals and titer ratio in study groups (GMR [95%CI]).

Parameter	18–59 Years	60–85 Years	Not Adjusted		Baseline-Corrected	
	GMT1	GMT2	GMR [95%CI]	*p* *	GMR [95%CI]	*p* **
B/Austria/1359417/2021 (B/Victoria lineage)-like virus	30.1 [23.9–37.9]	67.3 [42.1–108]	2.23 [1.33–3.74]	0.003	1.37 [0.88–2.15]	0.15
B/Phuket/3073/13 (B/Yamagata lineage)-like virus	49.7 [41.1–60.2]	25.5 [15–43.3]	0.51 [0.29–0.89]	0.02	0.74 [0.54–1.00]	0.05
A/Victoria/2570/2019 (H1N1)pdm09-like virus	109 [84.1–141]	67.3 [29.2–155]	0.62 [0.26–1.46]	0.26	0.91 [0.61–1.36]	0.61
A/Darwin/9/2021 (H3N2)-like virus	162 [133–197]	149 [79.5–280]	0.92 [0.78–1.77]	0.80	0.98 [0.69–1.41]	0.92

Notes: * Student’s *t*-test (for equal variances) or Welch’s test (for unequal variances) for log-transformed data was applied. ** ANCOVA was used with the inclusion of the antibodies baseline level and correction for heteroscedasticity of residuals.

**Table 7:** Incidence of local and systemic reactions.

Reactions	18–59 Years	60–85 Years	*p*
	*n* = 124	*n* = 20	
Local post-vaccination reactions	30 (24.2%)	4 (20%)	0.78
Systemic reactions	14 (11.3%)	3 (15%)	0.71
Combination of local and systemic reactions	5 (4%)	-	1.00

## Data Availability

Deidentified data presented in this manuscript will be made available 6 months after publication on reasonable request by email to the corresponding author for research purposes.
